# *Nuevo Amanecer‐II*: Results of a randomized controlled trial of a community‐based participatory, peer‐delivered stress management intervention for rural Latina breast cancer survivors

**DOI:** 10.1002/pon.5481

**Published:** 2020-08-07

**Authors:** Anna María Nápoles, Jasmine Santoyo‐Olsson, Anita L. Stewart, Carmen Ortiz, Cathy Samayoa, Alma Torres‐Nguyen, Helen Palomino, LaVerne Coleman, Aday Urias, Nayeli Gonzalez, Silvia Araceli Cervantes, Vicken Y. Totten

**Affiliations:** ^1^ Division of Intramural Research National Institute on Minority Health and Health Disparities, National Institutes of Health Bethesda Maryland USA; ^2^ Division of General Internal Medicine, Department of Medicine University of California San Francisco (UCSF) San Francisco California USA; ^3^ Center for Aging in Diverse Communities University of California San Francisco San Francisco California USA; ^4^ Institute for Health & Aging University of California San Francisco San Francisco California USA; ^5^ Círculo de Vida Cancer Support and Resource Center San Francisco California USA; ^6^ Health Equity Research Lab, Department of Biology San Francisco State University San Francisco California USA; ^7^ Kaweah Delta Health Care District Visalia California USA; ^8^ Cancer Resource Center of the Desert El Centro California USA; ^9^ WomenCARE/Entre Nosotras, Family Service Agency of the Central Coast Park California USA

**Keywords:** cancer, breast cancer survivors, community‐based participatory research, Latino/Hispanic, psychological distress, psycho‐oncology, quality of life, randomized controlled trial, rural communities, stress management

## Abstract

**Objective:**

We report results of a community‐based multisite, randomized controlled trial of *Nuevo Amanecer (NA‐II)*, a 10‐week stress management program for rural, low literacy Latina breast cancer survivors.

**Methods:**

Trained peers delivered *NA‐II* to Spanish‐speaking Latinas with non‐metastatic breast cancer in three rural communities. Women were randomized to receive the program immediately or wait 6 months. Assessments were conducted at baseline, 3 months, and 6 months. Primary outcomes were breast cancer‐specific quality of life domains; secondary outcomes included general distress symptoms and stress management skills. Intention‐to‐treat analyses using repeated‐measures linear regression models estimated changes in slope between groups.

**Results:**

Of 153 participants (76 randomized to intervention, 77 to control group), 92% were retained at 6 months. Mean age was 54.8 years (SD = 10.5); 80% had less than high school education. There were no statistically significant treatment × time effects on quality of life. Compared to women in the control group, intervention group women reported greater improvements in *anxiety* at 6 months (−0.20 vs −0.02, *P* = .049; range 0‐4) as well as three stress management skills: *relaxation* at 3 months (+0.98 vs −0.07, *P* < .0001; range 0‐4) and 6 months (+0.82 vs +0.04, *P* < .001), *awareness of tension* at 3 months (+0.31 vs −0.19, *P* < .01; range 0‐4) and 6 months (+0.29 vs −0.11, *P* < .05), and *coping confidence* at 3 months (+0.12 vs −0.23, *P* < .01; range 0‐4).

**Conclusions:**

Stress management programs delivered by trained peers in rural community settings can reduce anxiety and improve stress management skills among Latina breast cancer survivors.

## 1 BACKGROUND

Rural Latina breast cancer survivors are particularly vulnerable, experiencing poverty, legal status issues, language barriers, and limited health and breast cancer care, transportation, education, and childcare.^1^, ^2^, ^3^, ^4^ Additionally, Latina breast cancer survivors in the U.S. who live near the U.S.‐Mexico border have unique problems due to bi‐national health care seeking, poor cancer care coordination, regional shortages of cancer specialists, and financial hardship.^5^ Rural Latino cancer survivors reported language discordance with clinicians, unsatisfactory communication via medical interpreters, and lack of clarity of clinician communication.^6^ Consequently, they are at elevated risk of chronic stress and poor health‐related quality of life.

Cognitive‐behavioral stress management interventions have been demonstrated in randomized controlled trials (RCTs) to: increase relaxation and positive affect; reduce anxiety, depressive symptoms, and serum cortisol; and improve quality of life among breast cancer survivors.^7^, ^8^ Furthermore, such interventions may improve survival and reduce risk of recurrence among women with non‐metastatic breast cancer.^8^, ^9^

Latina breast cancer survivors report higher rates of anxiety, depression, fear of recurrence, fatigue, pain, and worse health‐related quality of life than their White counterparts.^10^, ^11^, ^12^ At least five randomized controlled trials (RCTs) have tested psychosocial interventions focused on stress reduction among Latinas.^10^, ^13^, ^14^, ^15^, ^16^ However, RCTs testing psychosocial interventions among *rural* Latina breast cancer survivors are needed. One small study (N = 14) tested a cognitive‐behavioral stress management program in patients with varied types of cancer in rural New England settings.^17^

Our original *Nuevo Amanecer (NA)* program was designed for newly diagnosed Latina breast cancer survivors in urban settings and successfully improved several quality of life domains and decreased breast cancer concerns and depressive and bodily symptoms.^18^ We adapted *NA* for Latina breast cancer survivors living in rural settings and expanded the content to include self‐care into survivorship to create *NA‐II*. In this paper, we report the results of a community‐based multisite, RCT of *NA‐II* in settings serving rural Latinas with breast cancer.

## 2 METHODS

We provide a brief description of the *NA‐II* program and study. Detailed descriptions of study settings, design, recruitment, adaptations, and program content are described elsewhere.^19^

### 2.1 Settings

The study was conducted in three California sites serving Spanish‐speaking Latina breast cancer survivors in rural areas that rely on agribusiness. Two were community‐based organizations (CBOs) and one was a community hospital. The sites/community partners were Cancer Resource Center of the Desert (CRCD) (Imperial Valley), WomenCARE/Entre Nosotras (Watsonville/Salinas), and Kaweah Delta Health Care District (KDHCD)(Tulare County in the Central Valley). CRCD is the only non‐profit organization in the Imperial Valley providing Spanish‐language comprehensive cancer support and patient navigation services. WomenCARE is a cancer support program of the Family Service Agency of the Central Coast (a mental health services provider) and Entre Nosotras is the Spanish‐language arm of WomenCARE. KDHCD is the only Visalia hospital offering comprehensive health care services, including community health workers who provide health education.

Community‐based participatory research approaches were employed from study conceptualization to dissemination of results. Numerous individuals from each community setting were involved including field staff who were employees of the partner sites (project director, at least two recruiters, and at least two interventionists called *compañeras* at each site), clinicians, survivors, and organizational leaders. Ongoing participation of community members occurred through community consultations, weekly calls and monthly videoconferencing or in‐person meetings with field staff, and review and pretesting of materials by community members. Trained community field staff implemented the study. A lead CBO and partner in the first RCT, *Circulo de Vida* Cancer Support and Resource Center (CDV), provided clinical supervision of field staff and assisted with training and implementation. CDV is a San Francisco Bay Area Spanish language cancer support services provider.

### 2.2 Study design

This was a 6‐month RCT to assess the effectiveness of *NA‐II* in improving breast cancer‐specific quality of life (primary outcomes) at 3 and 6 months.^19^ Secondary outcomes included four general distress symptoms, and attainment of four stress‐management skills. We compared the 10‐week *NA‐II* intervention group to a wait‐list control group. The University of California San Francisco Institutional Review Board (IRB) (protocol #16‐18 737) and the KDHCDIRB (protocol # 20160434006) approved the study. UCSF was the IRB of record for the other two sites. Written informed consent was obtained. The study conforms to the U.S. Federal Policy for the Protection of Human Subjects and is registered at http://www.ClinicalTrials.gov (NCT02931552).

### 2.3 Participants

Inclusion criteria, which were broad because this was an effectiveness trial, consisted of: (1) Spanish‐speaking Latina (self‐identified ethnicity); (2) diagnosis of Stage 0 to IIIC primary breast cancer; and (3) residing in the selected rural California communities (Imperial, Tulare, or Santa Cruz/Monterey counties) served by our community partners. Exclusion criteria were terminal illness, metastatic breast cancer diagnosis, or plans to move out of the area in six months.

### 2.4 Recruitment

Eligibility screening and recruitment were conducted with a bilingual script by trained bilingual Latinas employed by the community‐based partners. A project director at each site supervised recruiters. For the two CBO sites, recruiters identified potentially eligible women through intake records or outreach and education activities. KDHCD identified potentially eligible women through electronic health records and then mailed them an initial contact letter on their stationery, a study information sheet, and a prepaid refusal postcard to return to KDHCD. If no refusal postcard was received within two weeks, the site recruiter initiated telephone contact with the potential participant. Recruiters verified eligibility through medical or intake records.

### 2.5 Nuevo Amanecer‐II intervention

The *NA‐II* program was adapted from *NA* to expand its generalizability for rural, low literacy Spanish‐speaking Latina breast cancer survivors throughout survivorship. Adaptation processes for this trial are described in detail elsewhere.^19^, ^20^ Social cognitive theory was the conceptual framework used for *NA* and *NA‐II*.^21^ Adaptations for *NA‐II* were based on new formative research with survivors, advocates, patient navigators, medical social workers, and iterative consultations with community representatives from the three rural areas. Results led to expansion of the program from 8 to 10 sessions to accommodate more practice of skills and healthy lifestyles content, greater use of audio‐visuals, further simplification, and creation of a handout for family members providing information on the program.^20^

The *NA‐II* program included ten weekly modules which covered managing the impact of cancer, learning about breast cancer and survivorship, finding cancer information, getting support, identifying helpful and unhelpful thoughts, managing thoughts and mood, stress management techniques, managing activities that affect mood, healthy lifestyles, and goal‐setting. The program emphasized cognitive‐behavioral coping skills training, coaching, and modeling to actively manage stress and emotions. Core components consist of stress management skills training (eg, deep breathing, guided imagery), cognitive reframing (turning unhelpful thoughts into helpful thoughts), effective communication (with clinicians, family, friends), setting goals, and self‐regulation to achieve goals. Women were provided with general information on breast cancer survivorship (potential symptoms and side effects, survivorship care planning) and healthy lifestyles (nutrition and physical activity).

Potential *compañeras* (interventionists) who were Spanish‐speaking breast cancer survivors and at least three years post‐diagnosis with no active recurrence were identified by the community sites. The community Co‐PI, a clinical psychologist at CDV, interviewed candidates using a structured protocol, selecting women who demonstrated excellent interpersonal communication skills, compassion, cultural awareness, and having processed their personal breast cancer experience.

*Compañeras* participated in a 3‐day training (three eight‐hour consecutive days) conducted in Spanish by the PI, Co‐PI, and the Co‐PI's clinical director. Interactive didactic sessions covered psychosocial reactions to breast cancer among Latinas, the theoretical basis of the program, and hands‐on review of the modules with extensive demonstration and role modeling of cognitive‐behavioral stress management skills. *Compañeras* were trained to model skills for participants.^20^

*Compañeras* and participants were provided with a step‐by‐step Spanish‐language manual covering 10‐weekly sessions. Each week at participants’ homes, one 90‐minute module was presented in‐person using visuals and hands‐on exercises to teach and reinforce concepts and skills.

### 2.6 Assessments

Baseline, three‐month, and six‐month assessments corresponded with our aims of evaluating the 10‐week intervention soon after completion (three months) and retention of benefits after program termination (six months). Trained, community‐based recruiters conducted in‐person 60‐minute baseline assessments (in Spanish) in the participant's home or community partner office. Three‐ and six‐month assessments (30‐minute telephone surveys) were conducted by an experienced, bilingual research associate blinded to participants’ group assignment. Study data were managed using REDCap.^22^ Participants received $90 for three surveys.

#### 2.6.1 Measures

Primary outcomes were breast cancer‐specific quality of life measures. Secondary outcomes consisted of two types of measures: general distress symptoms and stress management skills. We describe the psychometric properties and descriptive statistics of measures in this sample (Table 1).

**TABLE 1:** *Nuevo Amanecer‐II* measures: descriptive statistics, internal‐consistency reliability, and item‐scale correlations (N = 153)

Measure (variable)	Direction of Score	# of Items	Range of Item‐scale Correlations	Alpha	Possible Range	Observed Range	Mean Scale Score (SD)
**Breast Cancer‐Specific Quality of Life** ^a^							
Physical well‐being	↑ = better	6	.48–.77	.84	0–24	2–24	17.4 (5.3)
Social/family well‐being	↑ = better	5	.48–.64	.79	0–20	0–20	13.6 (4.1)
Emotional well‐being	↑ = better	5	.35–.62	.74	0–20	2–20	14.4 (4.3)
Enjoyment of life (Functional well‐being)	↑ = better	4	.38–.62	.75	0–16	4–16	11.0 (2.9)
Breast cancer concerns	↑ = better	7	.14–.60	.62	0–28	8–28	17.4 (4.7)
Overall quality of life (FACT‐B total score)	↑ = better	27	NA	NA	0–108	33–108	73.7 (15.1)
**General Distress Symptoms**							
Depressive symptoms^b^	↑ = worse	8	.49–.62	.83	0–24	0–24	6.7 (5.3)
Perceived stress^c^	↑ = worse	10	.38–.73	.85	0–40	0–35	15.7 (7.3)
Anxiety^d^	↑ = worse	6	.62–.75	.88	0–4	0–3.8	0.70 (0.76)
Somatization^d^	↑ = worse	6	.35–.63	.70	0–4	0–3.6	0.70 (0.64)
**Stress Management Skills** ^**e**^							
Relaxation	↑ = better	2	.49	.66	0–4	0–4	2.0 (1.1)
Awareness of tension	↑ = better	3	.58–.63	.76	0–4	0–4	2.4 (0.97)
Assertiveness	↑ = better	3	.69–.81	.87	0–4	0–4	2.6 (1.1)
Coping confidence	↑ = better	5	.73–.83	.91	0–4	0–4	2.5 (0.91)

^a^ Breast cancer‐specific quality of life scales = FACT‐B scales.^23^

^b^ Depressive symptoms = PHQ‐8.^25^

^c^ Perceived stress = Perceived Stress Scale.^26^

^d^ Anxiety and somatization = Brief Symptom Inventory scales.^28^

^e^ Stress management scales = Measure of Current Status Part A (MOCS‐A).^29^

*Breast cancer‐specific quality of life* was assessed using the Functional Assessment of Cancer Therapy ‐ Breast (FACT‐B),^23^ in Spanish.^24^ The FACT‐B comprises five subscales on four dimensions of well‐being (physical, social/family, emotional, functional) and one assessing concerns about breast cancer, and a summary index (FACT‐B total score). Women were asked the extent to which statements applied to them during the prior 7 days with five response options ranging from 0 = not at all to 4 = very much. We used scales as modified in the original *NA* study based on results of psychometric analyses.^18^ We re‐labeled the FACT‐B *functional well‐being* scale as “*enjoyment of life*” because items pertain to this more general aspect of well‐being (eg, enjoy life, enjoy doing things, accepted illness).

FACT‐B scales were scored by summing item responses after reversing some items, with higher scores indicating better quality of life. Possible score ranges are: *physical well‐being*, 0 to 24; *social/family well‐being*, 0 to 20; *emotional well‐being*, 0 to 20; *enjoyment of life*, 0 to 16; and *breast cancer concerns*, 0 to 28. The total FACT‐B score (*overall quality of life*) was the sum of the five subscales (range 0‐108). Item‐scale correlations ranged from 0.35 to 0.77 and Cronbach alphas were ≥ 0.74 for all scales, except for *breast cancer concerns*, which had item‐scale correlations of 0.14 to 0.60 and Cronbach alpha = 0.62 (Table 1). We retained the *breast cancer concerns* scale since it is a part of the FACT‐B, a widely validated measure of breast cancer‐specific quality of life.

Secondary outcomes of general distress symptoms were assessed with four measures: *depressive symptoms*, *perceived stress*, *anxiety*, and *somatization*. *Depressive symptoms* were assessed with thePHQ‐8.^25^ Respondents reported the frequency with which they were bothered by the symptoms over the last 2 weeks on a 4‐point scale (0 = not at all, 1 = several days, 2 = more than half the days, and 3 = nearly every day). The score is calculated as the sum of the 8 items; scores range from 0 to 24; higher scores indicate more depressive symptoms. Item‐scale correlations ranged from 0.49 to 0.62 and Cronbach alpha was 0.83.

*Perceived stress* was assessed using the 10‐item Perceived Stress Scale (PSS Spanish version from the HCHS/SOL Sociocultural Ancillary Study).^26^, ^27^Scores range from 0 to 40; higher scores indicate more stress. Item‐scale correlations ranged from 0.38 to 0.73 and Cronbach alpha was 0.85.

*Anxiety* and *somatization* were assessed with two scales from the Brief Symptom Inventory (BSI).^28^ Women were asked how much each symptom distressed or bothered them during the past 2 weeks with response options ranging from 0 = not at all to 4 = extremely. Scores are calculated as the mean of non‐missing items (possible range 0‐4); higher scores indicate more anxiety or somatization. In our sample, item‐scale correlations were: *anxiety*, 0.62 to 0.75; *somatization*, 0.35 to 0.63. Cronbach alphas were: *anxiety*, 0.88; *somatization*, 0.70.

Stress management skills were assessed with the Measure of Current Status Part A (MOCS‐A).^29^The MOCS‐A comprises four subscales: *relaxation*, *awareness of tension*, *assertiveness*, and *coping confidence*. These skill areas were addressed in the intervention. Women are asked the extent to which they can do each technique when under stress (response choices: 0 = I cannot do this at all, 1 = I can do this just a little bit, 2 = I can do this fairly well, 3 = I can do this very well, and 4 = I can do this extremely well). We used a Spanish translation from a study of Spanish‐speaking prostate cancer survivors.^30^, ^31^ Non‐missing items are averaged with scores ranging from 0 to 4; higher scores indicate greater confidence in skills.

The 2‐item *relaxation* scale assessed women's ability to use relaxation techniques (muscle relaxation, mental imagery) to reduce tension. The 3‐item *awareness of tension* scale measures their ability to recognize bodily tension, stressful situations, and when they are becoming tense. The 3‐item *assertiveness* scale measures the extent to which women are able to ask for help/support when needed and can clearly express their needs. The 5‐item *coping confidence* scale measures their ability to re‐examine their thoughts to gain a new perspective, decide how to cope with problems, come up with emotionally balanced thoughts, and choose the best coping responses. Item‐scale correlations were: *relaxation*, 0.49; *awareness of tension*, 0.58 to 0.63; *assertiveness*, 0.69 to 0.81; *coping confidence*, 0.73 to 0.83. Cronbach alphas were: *relaxation*, 0.66; *awareness of tension*, 0.76; *assertiveness*, 0.87; *coping confidence*, 0.91.

Descriptive characteristics included age, language acculturation,^32^ education, health insurance, employment status, financial hardship, ethnicity, national origin, U.S. or foreign‐born, marital status, health care utilization, and presence of other chronic medical conditions. We assessed self‐rated health using the standard item (In general, would you say your health is…poor, fair, good, very good, excellent) and a new parallel item focusing on mental health (In general, would you say your mental health is…), with the same responses. Breast cancer characteristics verified through medical records review included cancer type, stage at diagnosis, type of surgery, and type of treatment.

#### 2.6.2 Participant adherence and fidelity to intervention

To assess participant adherence, *compañeras* completed a structured program tracking form after each session that included the session date, location, and duration. Program adherence was defined as having completed at least 7 of 10 sessions.

For each *compañera*, at least one intervention session was selected based on convenience, for direct observation for fidelity assessment by the Co‐PI. Of the 7 *compañeras*, 2 were observed twice. Long travel distances (up to 10 hours) and difficulty coordinating participant, *compañera* and Co‐PI schedules prevented observation of more visits. Using structured rating scales, the Co‐PI rated interventionists’ compliance with six dimensions on a scale from 1 = not at all to 4 = constantly: extent to which they followed the manual content for that session; explained concepts in easy‐to‐understand language; checked with participant to ensure comprehension; used caring/supportive communication; modeled skills for participants; and provided feedback to participants on skills. Sessions were audiotaped, and reviewed and rated also by two research associates, using the same rating scale. Scores on the six dimensions were calculated as the mean score across coders for each session.

#### 2.6.3 Randomization

Randomization was stratified by recruitment site. Before initiating recruitment, stratum‐specific sequential identification numbers were generated and randomly preassigned in blocks of random sizes. The individual was the unit of randomization with 1:1 allocation to experimental groups. Women were randomized to the *NA‐II* intervention or a wait‐list control group. After the baseline assessment, the recruiter gave the participant a sealed envelope with the next sequential identification number from her stratum indicating to which experimental group she had been assigned. Control group participants received usual care until after the 6‐month assessment at which time they were offered the intervention.

#### 2.6.4 Statistical analysis

A power analysis conducted to establish our enrollment target of 140 women assumed 80% power, two‐tailed α = 0.05, and 90% retention. Intention‐to‐treat analyses focused on treatment × time interaction effects at baseline, three and 6 months, for the primary outcomes of FACT‐B subscale scores. The study was powered for a minimal detectable effect of the proposed design of *d* = 0.45as estimated by simulation.

Likelihood‐based model estimation assumed outcome responses were missing at random.^33^ Explanatory variables included an intervention group indicator, a categorical time indicator, and a treatment × time interaction variable. Custom contrasts estimated differences between treatment groups at each assessment, as well as 2 treatment × time interactions: one examining the change from baseline to 3 months (immediately after intervention) and one examining the change from baseline to 6 months. We compared experimental groups on primary outcomes of *breast cancer‐specific quality of life* scales and secondary outcomes of general distress symptoms (*depressive symptoms*, *perceived stress*, *anxiety*, and *somatization*), and stress management skills(*relaxation*, *awareness of tension*, *assertiveness*, *coping confidence*).

## 3 RESULTS

**FIGURE 1:**
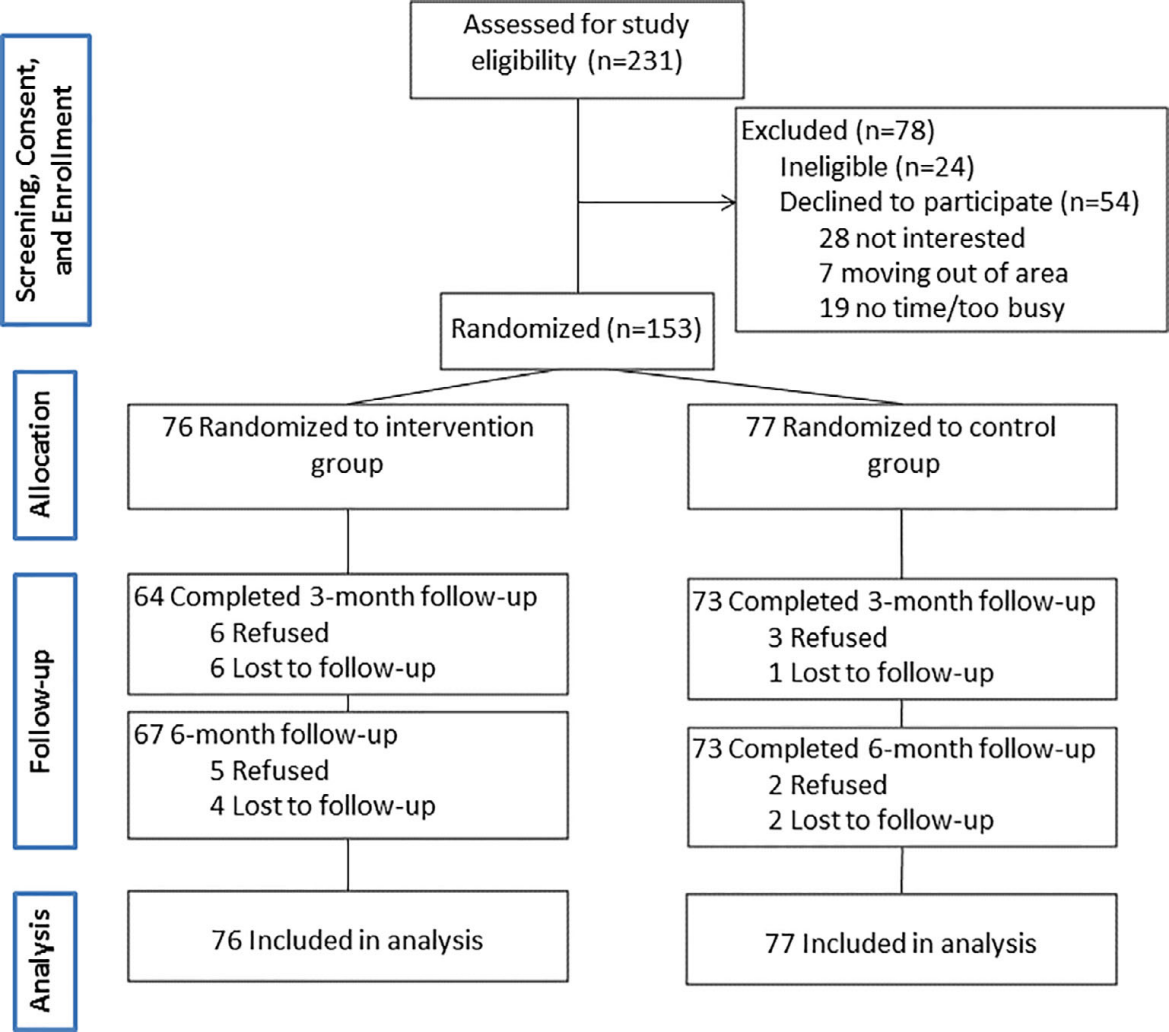
Flow of participants from screening to completion of follow‐up assessment, *Nuevo Amanecer‐II* study, California, September 2016 to March 2018

The RCT was conducted between September 2016 and October 2018. Figure 1 shows the Consolidated Standards of Reporting Trials (CONSORT) participant flow diagram. We invited 231 women; 24 were ineligible and 54 refused. We randomly assigned the final sample of 153 women to the intervention (n = 76) or control group (n = 77) between September 2016 and March 2018. Follow‐up assessments occurred from February 2017 through October 2018.

The overall sample had a mean age of 54.8 years (SD = 10.5, range 28‐88) (Table 2). Eighty percent had less than a high school education, most had public insurance only (75%), the majority were unemployed (82%), and almost half (48%) reported financial hardship in the past year. Nearly all (97%) reported being Mexican and 82% had limited English proficiency. Two‐thirds (66%) was married/living with a partner. Slightly less than half were in poor or fair health, and more than a third reported having poor or fair mental health.

**TABLE 2:** Baseline characteristics of Spanish‐speaking rural Latina breast cancer survivors participating in the *Nuevo Amanecer‐II* study, California, September 2016 to March 2018

Characteristics	Intervention Group (n = 76)	Control Group (n = 77)	*P*‐values	Total Sample (n = 153)
Age in years (mean, SD) (range 28–88)	55.7 (10.4)	53.8 (10.5)	0.313	54.8 (10.5)
Education (n, %)			0.022	
<sixth grade	38 (51)	22 (29)		60 (39)
sixth grade to <high school	25 (33)	38 (49)		63 (41)
High school graduate or more	12 (16)	17 (22)		29 (19)
Health insurance (n, %)			0.324	
Any private	14 (18)	22 (29)		36 (24)
Public insurance only	60 (79)	54 (70)		114 (75)
None	1 (1)	0 (0)		1 (1)
Do not know	1 (1)	1 (1)		2 (1)
Employed full or part time (% yes)	14 (18)	14 (18)	0.970	28 (18)
Any financial hardship in past year (% yes)	38 (50)	36 (47)	0.688	74 (48)
Ethnicity (n, %)			0.135	
Mexican	75 (99)	74 (96)		149 (97)
Central America	1 (1)	0 (0)		1 (1)
Other	0 (0)	3 (4)		3 (2)
Limited English proficiency	64 (85)	60 (78)	0.239	124 (82)
Married or living with partner (% yes)	49 (64)	52 (68)	0.690	101 (66)
Poor or fair self‐rated health (n, %)	41 (54)	29 (38)	0.043	70 (46)
Poor or fair self‐rated mental health (n, %)	28 (38)	26 (34)	0.602	54 (36)
				
**Breast Cancer Characteristics** (n, %)				
Type of breast cancer			0.721	
DCIS	6 (8)	9 (12)		15 (10)
Invasive	59 (78)	58 (75)		117 (76)
Inflammatory	7 (9)	8 (10)		15 (10)
Missing	4 (5)	2 (3)		6 (4)
Stage at Diagnosis			0.802	
0	5 (7)	3 (4)		8 (5)
1	24 (32)	21 (27)		45 (29)
2	27 (36)	28 (36)		55 (36)
3	13 (17)	14 (18)		27 (18)
Missing	7 (9)	11 (14)		18 (12)
Surgical Treatment			0.519	
Breast conserving	39 (51)	37 (48)		76 (48)
Mastectomy	34 (45)	38 (49)		72 (47)
No surgical treatment	0 (0)	1 (1)		1 (1)
Missing	3 (4)	1 (1)		4 (3)
Adjuvant Treatment			0.623	
Radiotherapy and chemotherapy	48 (63)	43 (56)		91 (59)
Radiotherapy only	12 (16)	16 (21)		28 (18
Chemotherapy only	12 (16)	10 (13)		22 (14)
No adjuvant treatment	3 (4)	7 (9)		10 (7)
Missing	1 (1)	1 (1)		2 (1)
Years since most recent diagnosis (mean, SD)	2.54 (3.6)	2.51 (2.8)	0.950	2.5 (3.2)
				
**Breast Cancer‐Specific Quality of Life** ^a^(mean, SD)				
Physical well‐being (scale 0–24)	17.53 (5.16)	17.21 (5.51)	0.706	17.4 (5.3)
Social/family well‐being (scale 0–20)	13.11 (4.01)	14.12 (4.10)	0.128	13.6 (4.1)
Emotional well‐being (scale 0–20)	14.13 (4.36)	14.64 (4.30)	0.472	14.4 (4.3)
Enjoyment of life (functional well‐being, scale 0–16)	10.86 (2.81)	11.10 (2.96)	0.608	11.0 (2.9)
Breast cancer concerns (scale 0–28)	17.20 (4.69)	17.50 (4.82)	0.692	17.4 (4.7)
Overall quality of life (scale 0–108)	72.90 (14.46)	74.55 (15.71)	0.501	73.7 (15.1)
**General Distress Symptoms** (mean, SD)				
Depressive symptoms (scale 0–24)^b^	6.79 (5.06)	6.65 (5.48)	0.870	6.7 (5.3)
Perceived stress (scale 0–40)^c^	15.51 (7.63)	15.95 (7.08)	0.715	15.7 (7.3)
Anxiety (scale 0–4)^d^	0.72 (0.73)	0.68 (0.80)	0.722	0.70 (0.76)
Somatization (scale 0–4)^d^	0.67 (0.60)	0.73 (0.67)	0.538	0.70 (0.64)
**Stress Management Skills** ^e^ (mean, SD)				
Relaxation (scale 0–4)	1.78 (1.13)	2.13 (1.11)	0.053	2.0 (1.1)
Awareness of tension (scale 0–4)	2.32 (0.88)	2.49 (1.05)	0.283	2.4 (0.97)
Assertiveness (scale 0–4)	2.59 (1.11)	2.49 (1.22)	0.579	2.6 (1.1)
Coping confidence (scale 0–4)	2.45 (0.80)	2.53 (1.01)	0.607	2.5 (0.91)

^a^ Breast cancer‐specific quality of life scales = FACT‐B scales.^23^

^b^ Depressive symptoms = PHQ‐8.^25^

^c^ Perceived stress = Perceived Stress Scale.^26^

^d^ Anxiety and somatization = Brief Symptom Inventory scales.^28^

^e^ Stress management scales = Measure of Current Status Part A (MOCS‐A).^29^

**TABLE 3:** Quality of life, symptoms of distress, and coping skills among Spanish‐speaking Latinas with breast cancer, by treatment group at baseline, 3 months, and 6 months: *Nuevo Amanecer‐II* study, California, September 2016 to March 2018

	Intervention Mean (SD)	ControlMean (SD)	*P*‐value
**Breast Cancer‐Specific Quality of Life** (higher score = better quality of life)^a^			
Physical well‐being (scale 0–24)			
Baseline	17.53 (5.16)	17.21 (5.51)	0.706
3 months	18.78 (4.26)	18.36 (4.88)	0.283
6 months	19.12 (3.98)	18.73 (4.34)	0.445
Treatment × time interaction (0–3 months)			0.455
Treatment × time interaction (0–6 months)			0.746
Social/family well‐being (scale 0–20)			
Baseline	13.11 (4.01)	14.12 (4.10)	0.128
3 months	13.33 (3.59)	13.48 (4.22)	0.946
6 months	11.76 (4.24)	12.45 (4.57)	0.443
Treatment × time interaction (0–3 months)			0.138
Treatment × time interaction (0–6 months)			0.559
Emotional well‐being (scale 0–20)			
Baseline	14.13 (4.36)	14.64 (4.30)	0.472
3 months	15.33 (3.71)	15.10 (4.03)	0.624
6 months	15.30 (3.56)	15.18 (4.24)	0.815
Treatment × time interaction (0–3 months)			0.158
Treatment × time interaction (0–6 months)			0.298
Breast cancer concerns (scale 0–28)			
Baseline	17.20 (4.69)	17.50 (4.82)	0.692
3 months	17.78 (4.75)	18.93 (5.31)	0.289
6 months	19.15 (4.02)	19.38 (4.77)	0.871
Treatment × time interaction (0–3 months)			0.425
Treatment × time interaction (0–6 months)			0.792
Enjoyment of life (scale 0–16)			
Baseline	10.86 (2.81)	11.10 (2.96)	0.608
3 months	10.81 (2.71)	10.82 (3.01)	0.695
6 months	10.37 (3.03)	10.64 (3.27)	0.729
Treatment × time interaction (0–3 months)			0.324
Treatment × time interaction (0–6 months)			0.904
Overall quality of life (scale 0–108)			
Baseline	72.90 (14.46)	74.55 (15.71)	0.501
3 months	76.03 (13.70)	76.68 (17.55)	0.827
6 months	75.70 (13.35)	76.38 (17.29)	0.953
Treatment × time interaction (0–3 months)			0.230
Treatment × time interaction (0–6 months)			0.448
**General Distress Symptoms** (higher score = more distress)			
Depressive symptoms (scale 0–24)^b^			
Baseline	6.79 (5.06)	6.65 (5.48)	0.870
3 months	6.81 (5.31)	6.97 (5.12)	0.436
6 months	6.96 (5.62)	6.44 (5.15)	0.794
Treatment × time interaction (0–3 months)			0.244
Treatment × time interaction (0–6 months)			0.909
Perceived stress (scale 0–40)^c^			
Baseline	15.51 (7.63)	15.95 (7.08)	0.715
3 months	14.45 (6.63)	14.08 (7.35)	0.995
6 months	14.70 (6.14)	15.14 (6.28)	0.574
Treatment × time interaction (0–3 months)			0.633
Treatment × time interaction (0–6 months)			0.885
Anxiety (scale 0–4)^d^			
Baseline	0.72 (0.73)	0.68 (0.80)	0.722
3 months	0.63 (0.61)	0.65 (0.70)	0.833
6 months	0.52 (0.53)	0.70 (0.64)	0.094
Treatment × time interaction (0–3 months)			0.492
Treatment × time interaction (0–6 months)			**0.049**
Somatization (scale 0–4)^d^			
Baseline	0.67 (0.60)	0.73 (0.67)	0.538
3 months	0.64 (0.58)	0.72 (0.70)	0.202
6 months	0.48 (0.45)	0.65 (0.63)	**0.032**
Treatment × time interaction (0–3 months)			0.380
Treatment × time interaction (0–6 months)			0.117
**Stress Management Skills (higher score = better skills)** ^e^			
Relaxation skills (scale 0–4)			
Baseline	1.78 (1.13)	2.13 (1.11)	0.053
3 months	2.76 (0.72)	2.06 (0.96)	**<.0001**
6 months	2.60 (0.79)	2.17 (0.94)	**0.004**
Treatment × time interaction (0–3 months)			**<.0001**
Treatment × time interaction (0–6 months)			**0.0001**
Awareness of tension (scale 0–4)			
Baseline	2.32 (0.88)	2.49 (1.05)	0.283
3 months	2.63 (0.61)	2.30 (0.77)	**0.003**
6 months	2.61 (0.66)	2.38 (0.76)	**0.049**
Treatment × time interaction (0–3 months)			**0.002**
Treatment × time interaction (0–6 months)			**0.015**
Assertiveness (scale 0–4)			
Baseline	2.59 (1.11)	2.49 (1.22)	0.579
3 months	2.64 (0.91)	2.33 (0.88)	**0.015**
6 months	2.55 (0.91)	2.49 (0.96)	0.601
Treatment × time interaction (0–3 months)			0.113
Treatment × time interaction (0–6 months)			0.895
Coping confidence (scale 0–4)			
Baseline	2.45 (0.80)	2.53 (1.01)	0.607
3 months	2.57 (0.66)	2.30 (0.70)	**0.016**
6 months	2.50 (0.75)	2.46 (0.72)	0.676
Treatment × time interaction (0–3 months)			**0.008**
Treatment × time interaction (0–6 months)			0.345

^a^ Breast cancer‐specific quality of life scales = FACT‐B scales.^23^

^b^ Depressive symptoms = PHQ‐8.^25^

^c^ Perceived stress = Perceived Stress Scale.^26^

^d^ Anxiety and somatization = Brief Symptom Inventory scales.^28^

^e^ Stress management scales = Measure of Current Status Part A (MOCS‐A).^29^

The greatest proportion were diagnosed at stage II, with equal proportions of women receiving breast conserving vs mastectomy surgery and the majority receiving radiotherapy and chemotherapy. Average time since diagnosis was 2.5 years (SD = 3.2).

We found no significant baseline differences between intervention and control groups on demographics, clinical characteristics, quality of life, or distress outcomes, except for educational attainment and self‐rated health, with the intervention group tending to be slightly better educated and more likely to report poor or fair health.

Baseline levels of breast cancer‐specific quality of life were low, indicating poor quality of life (Table 2). In general, scores on *anxiety*, *somatization*, *depressive symptoms*, and *perceived stress* were low, indicating fairly low levels of general distress symptoms. Breast cancer self‐management skills were fair (2.0‐2.6 on a 0‐4 scale). There were no significant differences between intervention and control groups at baseline on quality of life, general distress, or stress management skills.

A large percentage (86%) of the intervention group completed at least 7 of 10 weekly sessions (9% completed 1 to 6 sessions, and 5% completed no sessions). Six‐month retention was 88% for the intervention group and 95% for the control group (92% overall). Mean fidelity ratings were: 4.5, extent followed manual; 4.4, used easy‐to‐understand language; 3.9, checked comprehension; 4.4, used caring/supportive communication; 3.8, modeled skills; and 3.9, provided feedback.

*Primary outcomes: breast cancer‐specific quality of life*. There were no statistically significant treatment × time interaction effects on quality of life outcomes at 3 or 6 months (Table 3).

**FIGURE 2:**
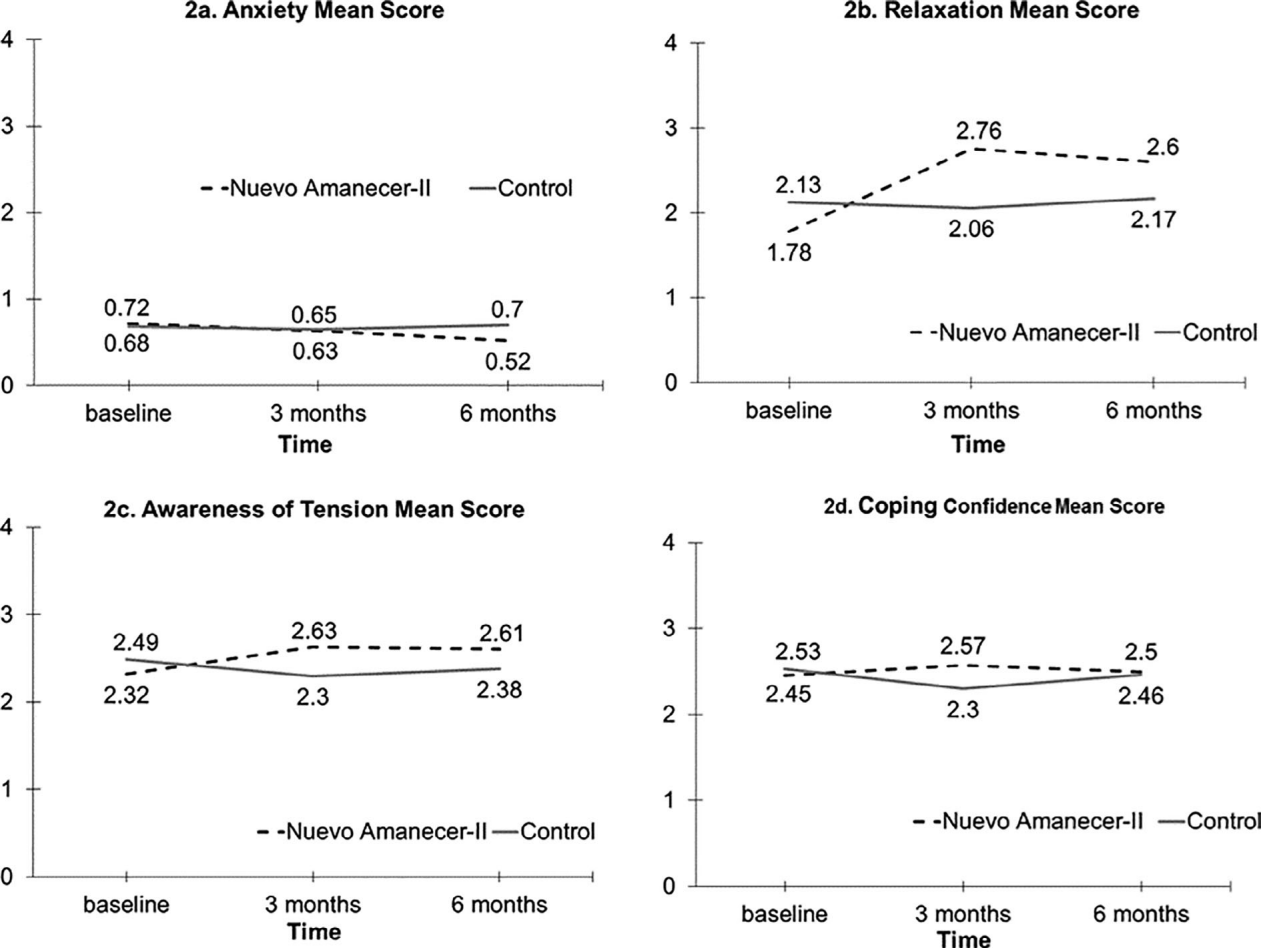
Anxiety and coping skills outcomes, *Nuevo Amanecer‐II* study, California, September 2016 to March 2018

*Secondary outcomes: general distress symptoms*. From baseline to 6 months (3 months post‐intervention), we found significant treatment × time interaction effects on *anxiety* (*P* = .049), with the intervention group reporting greater improvement than the control group (−0.20 vs −0.02; range 0‐4) (Figure 2). At 6 months, scores on *somatization* in the intervention group were significantly lower than in the control group (0.48 vs 0.65; range 0‐4; *P* < 0.05). However, there were no significant treatment × time interaction effects for *somatization*. There were no significant treatment × time effects on *depressive symptoms* or *perceived stress*.

*Secondary outcomes: stress management skills* (range 0‐4). The effects of the intervention on stress management skills varied. For *relaxation*, we observed significant treatment × time interaction effects at 3 months (*P* < 0.0001) and 6 months (*P* < 0.001). At 3 months, women in the intervention group experienced greater improvement in their relaxation skills than women from the control group (+0.98 vs −0.07). Positive effects on relaxation skills in the intervention group were maintained (+0.82) at 6 months (Figure 2).

For *awareness of tension*, we observed significant treatment × time interaction effects at 3 months (*P* < 0.01) and 6 months (*P* < 0.05). At 3 months, intervention group women experienced greater improvement in *awareness of tension* than the control group (+0.31 vs −0.19). Effects on *awareness of tension* in the intervention group were maintained (+0.29) at 6 months (Figure 2).

For *assertiveness*, we did not observe any treatment × time interaction effects, but 3‐month scores were significantly higher for the intervention (2.64) than control group (2.33) (*P* < 0.05).

For *coping confidence*, we observed significant treatment × time interaction effects at 3 months (*P* < 0.01). The intervention group improved their *coping confidence* from 2.45 to 2.57 (+0.12) at 3 months while the control group's scores declined from 2.53 to 2.30 (−0.23) (Figure 2).

## 4 DISCUSSION

In this study, we continued translation of our cognitive‐behavioral stress management program to be suitable for rural and urban Spanish‐speaking Latina breast cancer survivors through long‐term survivorship. We were able to reach and retain vulnerable, rural‐dwelling survivors. Compared to a wait‐list control group, women who received the intervention reported significant reductions in anxiety and improvements in several stress management skills, including their ability to relax at will, be aware of tension, and their confidence in coping with problems. However, there were no significant effects of the intervention on breast cancer‐specific quality of life.

The lack of effects on quality of life in this trial are inconsistent with our prior RCT among urban Latina breast cancer survivors. In that study, several quality of life domains improved including physical and emotional well‐being, breast cancer concerns, and overall quality of life, with no significant effects on anxiety. But in that study, all women were enrolled within 1 year of diagnosis and most within 3 months, whereas women in this trial were farther from diagnosis (within 2.5 years on average). Quality of life measures were breast cancer‐specific and may not have been as relevant in this study as more general measures of quality of life and distress.^18^ Also, possibly due to their longer time since diagnosis, baseline levels of quality of life among women in this study were better than those in our prior RCT, leaving less room for improvement. Our positive results with respect to anxiety in this trial are consistent with one other single‐arm trial in a small group of Latinas that found significant improvements in anxiety with an 8‐week mindfulness stress reduction program^14^.

Women in this study had several vulnerabilities including rural residence, low socioeconomic status, financial hardship, dependency on public insurance, high level of unemployment and disability, limited English proficiency, and immigrant status. For breast cancer survivors facing these vulnerabilities, providing a sense of confidence in their ability to cope with problems and reduce anxiety may be especially helpful. Since we did not see significant improvements in a general stress measure among the intervention group compared to the control group, it is possible that a more intensive or longer stress management program or a different type of program may be needed to effectively reduce stress in this especially vulnerable group. At a minimum, community partners increased their capacity to deliver evidence‐based stress management skills training.

### 4.1 Study limitations

Study limitations include that the sample consisted of Latina breast cancer survivors of Mexican origin only, living in rural California communities. However, many of the cultural values incorporated into the intervention, for example, familismo, spirituality, fatalism and respect are shared by Latinos broadly and cut across national origin. Careful attention was paid in the formative work for this and the prior RCT to ensure that the program met the needs of both rural and urban Latinas. Therefore, it is likely that the program would generalize to Latinas from other national origin groups in the U.S.

### 4.2 Clinical implications

Among breast cancer survivors, stress has been linked to immunological suppression and cancer progression, thus, reducing stress has important implications for preventing recurrence.^34^ There are significant disparities in self‐reported stress by socioeconomic status and race, and these stressors are known to have downstream effects on a range of psychological, neurobiological, and physiological processes and health behaviors.^35^ Providing vulnerable populations with the skills to reduce chronic stress in their lives could provide broader advantages to their health and well‐being given the cascade of ill‐effects and suffering that such stress can inflict.

## 5 CONCLUSIONS

A 2017 report by the National Academies of Sciences, Engineering, and Medicine provides a strong rationale for the community as the locus for confronting health inequities. They argue persuasively that solutions for health disparities involve local community action with a range of partners.^36^ Contributing to community infrastructure to address root causes of social and economic inequities and accompanying stress is a promising pathway to health equity,^36^ particularly for vulnerable cancer survivors.

## CONFLICT OF INTEREST

The authors declare no conflicts of interest.

## DISCLAIMER

The contents and views in this manuscript are those of the authors and should not be construed to represent the views of the National Institutes of Health.

## Data Availability

In accordance with the NIH Data Sharing Policy, de‐identified data collected as a part of this study and supporting documentation will be made available to other researchers who contact the Principal Investigator directly and complete a data transfer agreement.
